# Supplementary figures and images for: Single-Cell RNA Sequencing Reveals that the Switching of the Transcriptional Profiles of Cysteine-Related Genes Alters the Virulence of Entamoeba histolytica
Source: mSystems. 2020 Dec 22;5(6):e01095-20. doi: 10.1128/mSystems.01095-20 (PMC7762796; doi:10.1128/mSystems.01095-20)

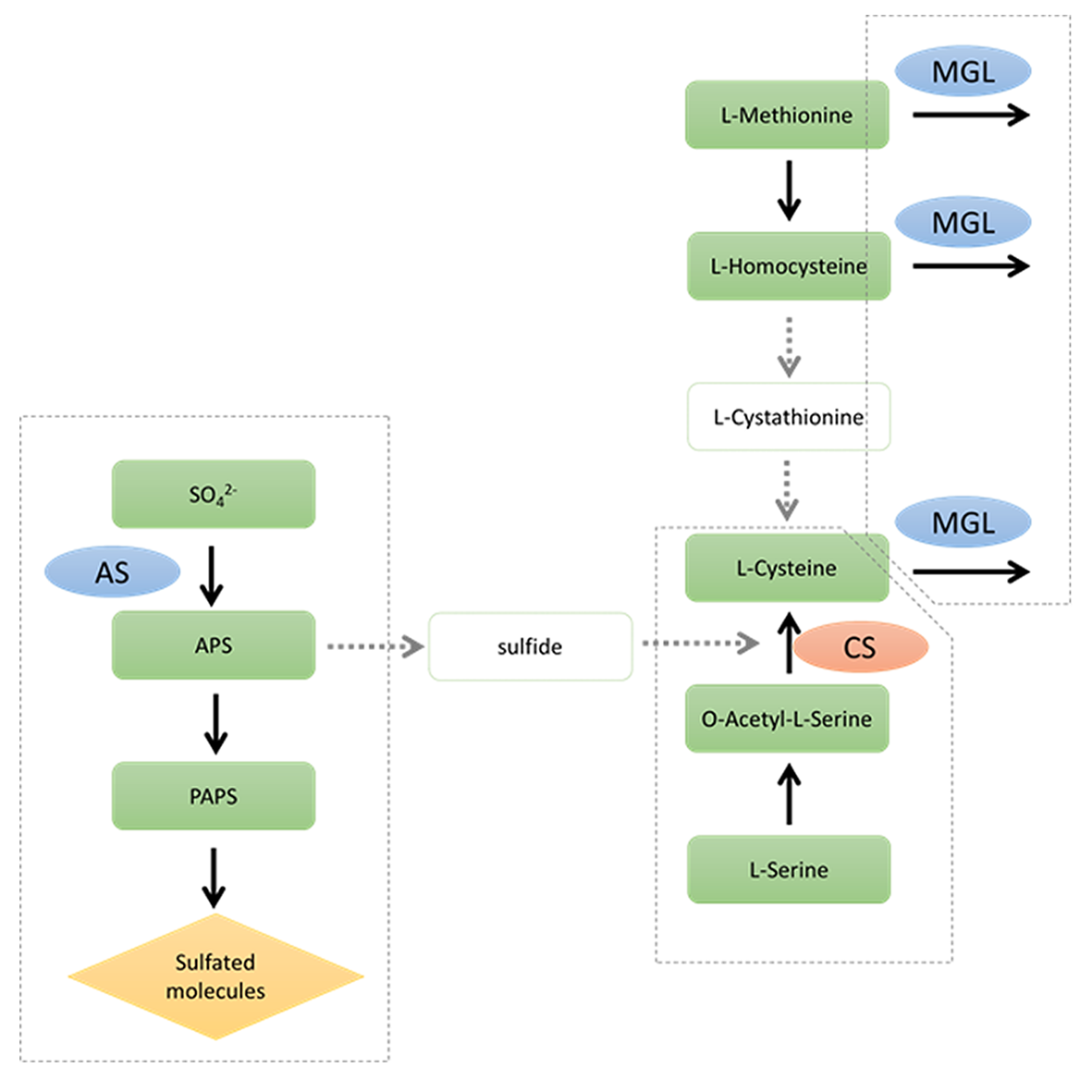

Supplement: FIG S1 [file mSystems.01095-20-sf001.tif]

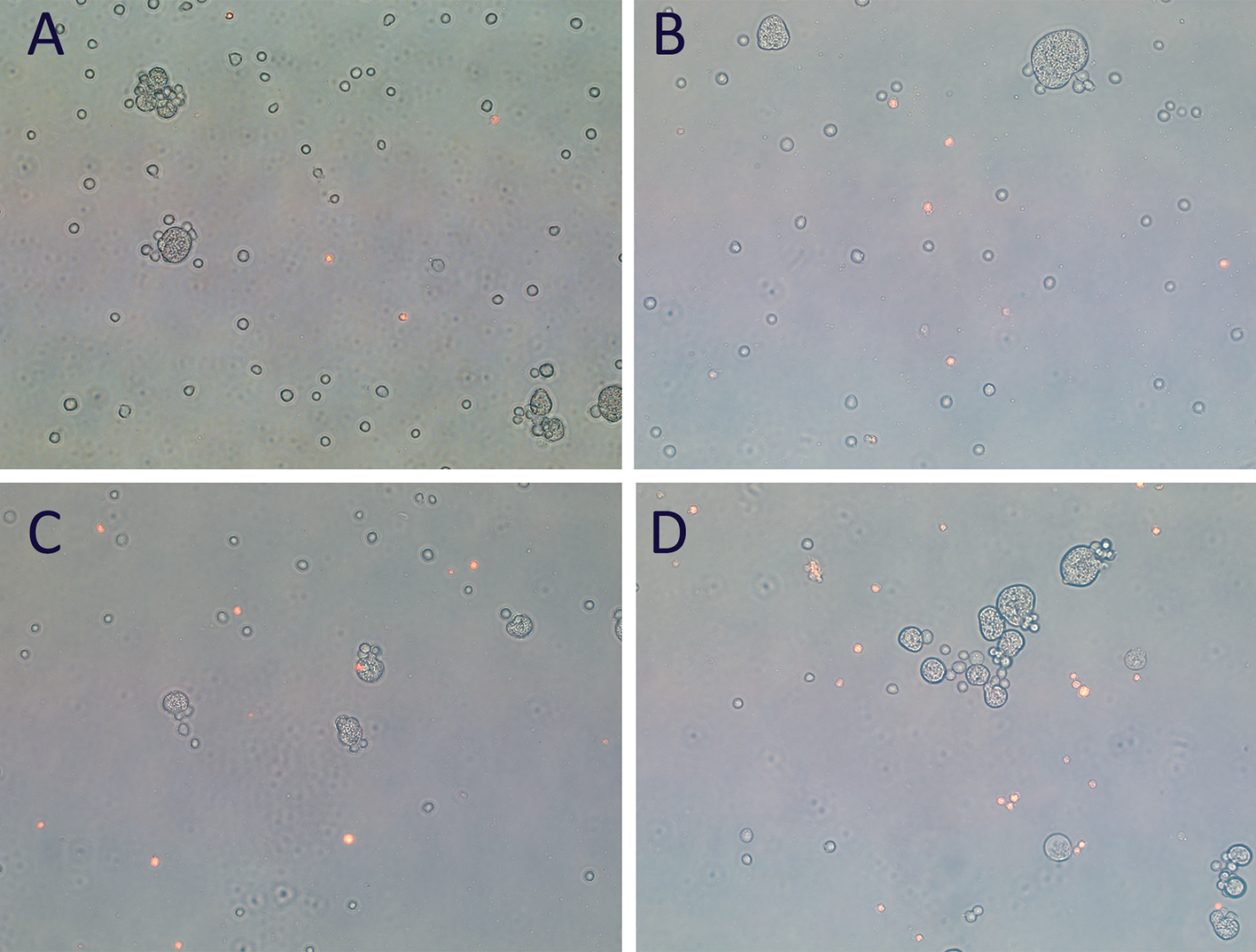

Supplement: FIG S2 [file mSystems.01095-20-sf002.tif]

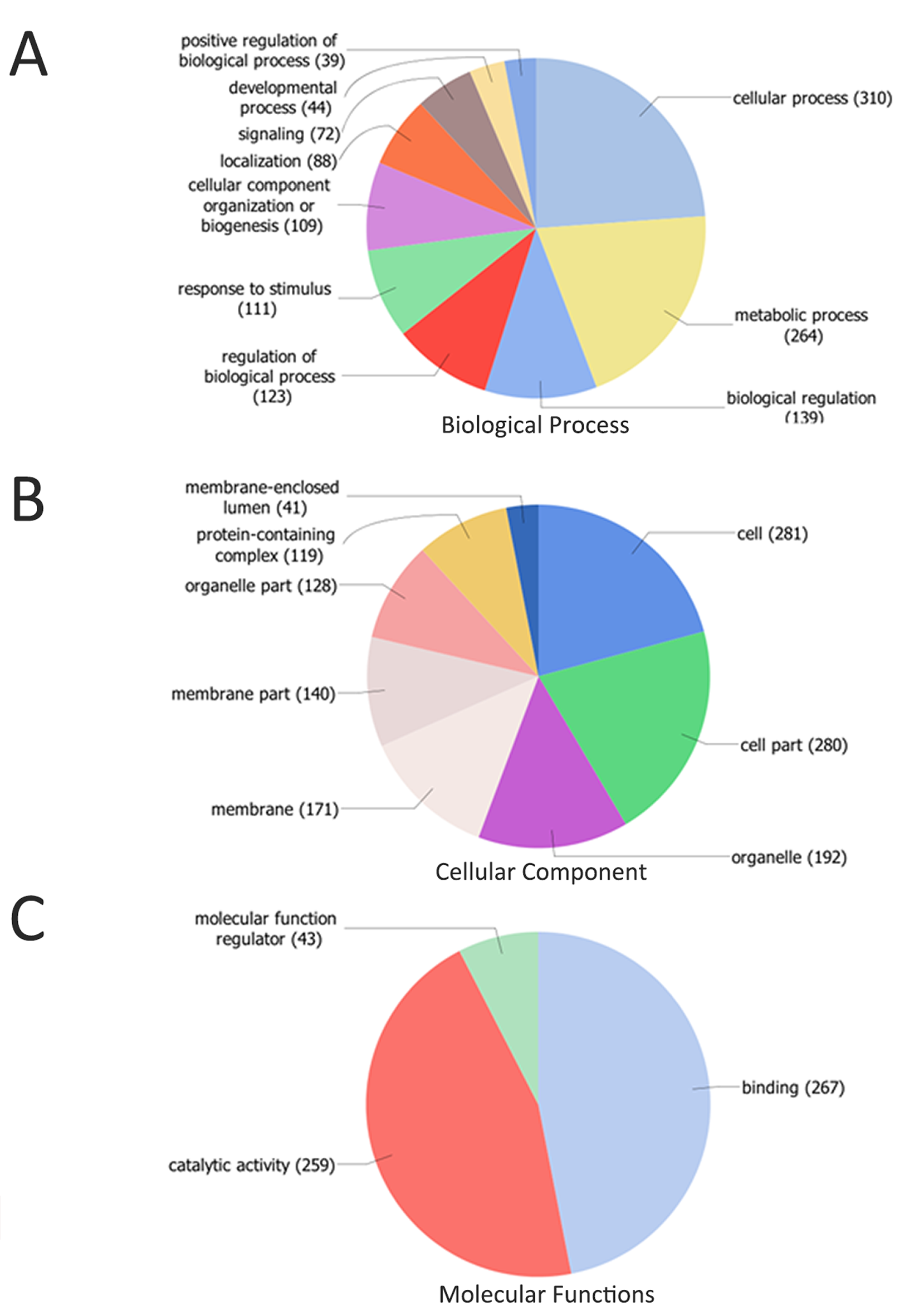

Supplement: FIG S3 [file mSystems.01095-20-sf003.tif]

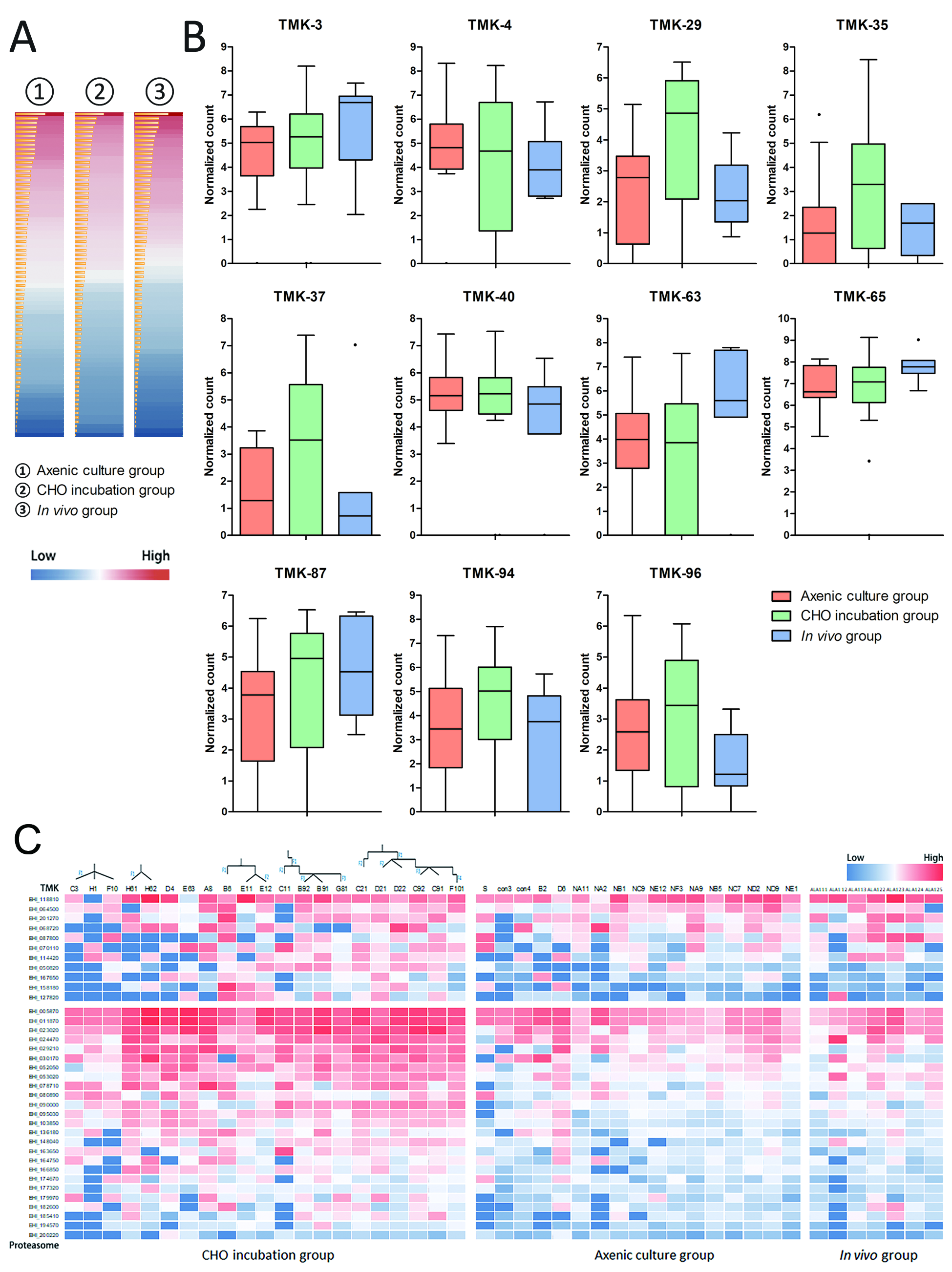

Supplement: FIG S4 [file mSystems.01095-20-sf004.tif]

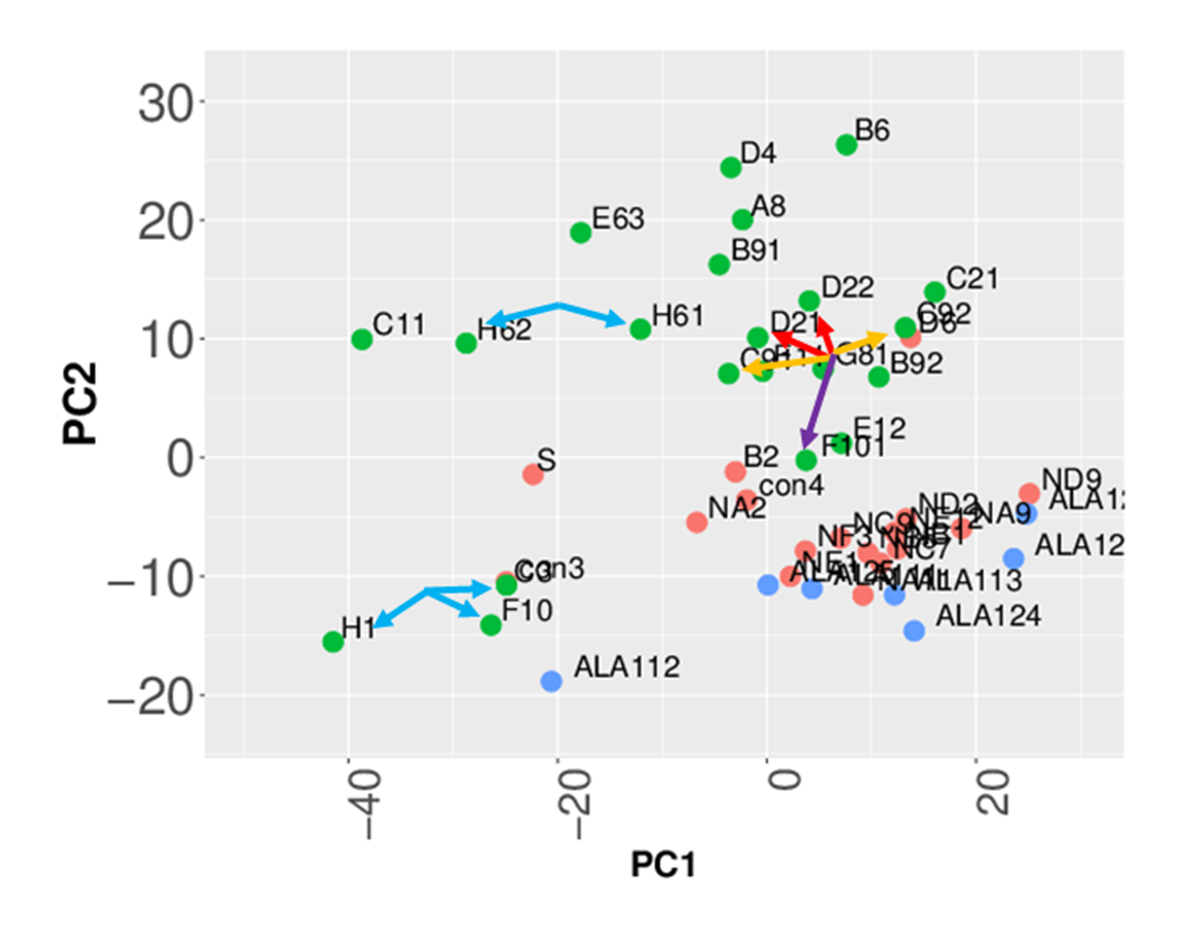

Supplement: FIG S5 [file mSystems.01095-20-sf005.tif]
