# Supplementary material for: Single-Cell RNA Sequencing Reveals that the Switching of the Transcriptional Profiles of Cysteine-Related Genes Alters the Virulence of Entamoeba histolytica
Source: mSystems. 2020 Dec 22;5(6):e01095-20. doi: 10.1128/mSystems.01095-20 (PMC7762796; doi:10.1128/mSystems.01095-20)
Supplement: TABLE S1 [file mSystems.01095-20-st001.docx]

| Identified proteins in iTRAQ quantitative proteomic analysis | | | | | | | | | | | |
| --- | --- | --- | --- | --- | --- | --- | --- | --- | --- | --- | --- |
| Gene | | Access number | | control | | 2h | | 4h | | 6h | |
| TMK1 | | EHI_065240 | | 1 | | 0.798676 | | 0.963809 | | 0.870127 | |
| TMK3 | | EHI_201270 | | 1 | | 1.407118 | | 1.147209 | | 1.486519 | |
| TMK6 | | EHI_135190 | | 1 | | 1.752009 | | 1.35308 | | 1.400977 | |
| TMK22 | | EHI_186990 | | 1 | | 1.150926 | | 1.477355 | | 1.067679 | |
| TMK37 | | EHI_127820 | | 1 | | 1.445491 | | 1.173691 | | 1.58645 | |
| TMK39 | | EHI_037140 | | 1 | | 1.181018 | | 1.104517 | | 1.122013 | |
| TMK40 | | EHI_064500 | | 1 | | 1.002631 | | 0.951161 | | 0.974608 | |
| TMK51 | | EHI_103420 | | 1 | | 1.115734 | | 1.011596 | | 1.043997 | |
| TMK52 | | EHI_148550 | | 1 | | 1.156093 | | 0.981654 | | 1.108845 | |
| TMK54 | | EHI_188110 | | 1 | | 1.041745 | | 1.027329 | | 0.950357 | |
| TMK59 | | EHI_097640 | | 1 | | 1.40985 | | 1.49662 | | 1.637329 | |
| TMK60 | | EHI_138750 | | 1 | | 1.111546 | | 1.335851 | | 1.321958 | |
| TMK65 | | EHI_118810 | | 1 | | 1.181414 | | 1.071808 | | 1.058269 | |
| TMK71 | | EHI_030420 | | 1 | | 2.857848 | | 2.187375 | | 2.591912 | |
| TMK94 | | EHI_070110 | | 1 | | 1.10171 | | 1.010362 | | 1.10664 | |
| Hgl | | EHI_012270 | | 1 | | 1.284502 | | 1.14089 | | 1.190362 | |
| Hgl | | XM_001913658 | | 1 | | 1.022467 | | 0.995609 | | 0.921773 | |
| Hgl3 | | L14815 | | 1 | | 1.107258 | | 0.994006 | | 1.148683 | |
| Igl-1 | |  | | 1 | | 1.236065 | | 1.052278 | | 1.044161 | |
| Igl-2 | | EHI_065330 | | 1 | | 1.261724 | | 1.120629 | | 1.035104 | |
| Lgl | |  | | 1 | | 1.169446 | | 1.115728 | | 1.080807 | |
| Lgl | | EHI_148790 | | 1 | | 0.893906 | | 0.817851 | | 0.841385 | |
| ameobapore A | |  | | 1 | | 0.183894 | | 0.289497 | | 0.249361 | |
| ameobapore B | |  | | 1 | | 0.349914 | | 0.518153 | | 0.267653 | |
| ameobapore C | |  | | 1 | | 0.747086 | | 0.952732 | | 1.053937 | |
| Cysteine proteinase 1 | |  | | 1 | | 1.34761 | | 1.334385 | | 1.585212 | |
| Cysteine proteinase 2 | |  | | 1 | | 0.988041 | | 0.93622 | | 1.061334 | |
| Cysteine proteinase 5 | |  | | 1 | | 1.309865 | | 1.287416 | | 1.137599 | |
| Cysteine proteinase | | EHI_050570 | | 1 | | 0.98761 | | 0.85144 | | 0.990593 | |
| Cysteine proteinase | | EHI_010850 | | 1 | | 0.934152 | | 0.909484 | | 0.921567 | |
| Proteasome subunit alpha type | | C4MA29_ENTHI | | 1 | | 0.951806 | | 0.987044 | | 1.015018 | |
| Proteasome regulatory subunit | | C4LVM7_ENTHI | | 1 | | 1.021306 | | 1.175372 | | 0.979038 | |
| Proteasome subunit alpha type-5 | | PSA5_ENTHI | | 1 | | 0.934545 | | 0.996172 | | 1.031005 | |
| Proteasome regulatory subunit | | C4M298_ENTHI | | 1 | | 1.00638 | | 1.021472 | | 1.097055 | |
| Proteasome beta subunit | | C4M4F3_ENTHI | | 1 | | 1.052666 | | 1.211698 | | 1.017728 | |
| Proteasome regulatory subunit | | C4LV41_ENTHI | | 1 | | 1.165258 | | 1.311013 | | 1.230752 | |
| Proteasome regulatory subunit | | C4M6T7_ENTHI | | 1 | | 0.958142 | | 1.012107 | | 0.990615 | |
| Proteasome subunit alpha type | | C4LSB1_ENTHI | | 1 | | 1.139415 | | 1.093868 | | 1.118976 | |
| Proteasome beta subunit | | C4LZL2_ENTHI | | 1 | | 0.966841 | | 1.089202 | | 1.05212 | |
| Proteasome subunit alpha type | | C4M854_ENTHI | | 1 | | 1.004886 | | 0.958689 | | 1.146055 | |
| Proteasome alpha subunit | | C4LY89_ENTHI | | 1 | | 1.10297 | | 1.091887 | | 1.129331 | |
| Proteasome regulatory subunit | | C4M2M2_ENTHI | | 1 | | 0.979204 | | 0.988388 | | 0.987544 | |
| Proteasome subunit alpha type | | C4M4A2_ENTHI | | 1 | | 1.108027 | | 1.114219 | | 1.034159 | |
| Proteasome alpha subunit | | C4M363_ENTHI | | 1 | | 1.003001 | | 0.966992 | | 1.035353 | |
| Proteasome subunit beta type | | C4LTI2_ENTHI | | 1 | | 1.23988 | | 1.136364 | | 1.286607 | |
| Proteasome regulatory subunit | | C4M451_ENTHI | | 1 | | 0.93504 | | 1.138713 | | 1.033115 | |
| Proteasome subunit beta type | | C4LSX4_ENTHI | | 1 | | 1.041406 | | 0.981362 | | 1.093508 | |
| Proteasome regulatory subunit | | C4M648_ENTHI | | 1 | | 0.949525 | | 0.933222 | | 0.977331 | |
| 26S proteasome non-ATPase regulatory subunit 14 | | A0A060N6N4_ENTHI | | 1 | | 0.937256 | | 0.984454 | | 0.987339 | |
| Proteasome regulatory subunit | | C4M9F1_ENTHI | | 1 | | 0.96486 | | 1.055925 | | 0.999464 | |
| Proteasome subunit beta type | | C4M0I9_ENTHI | | 1 | | 0.845262 | | 1.036726 | | 0.85832 | |
| Proteasome regulatory subunit | | C4M7M7_ENTHI | | 1 | | 0.814512 | | 0.937234 | | 0.583887 | |
| Proteasome subunit beta type | | C4LUD5_ENTHI | | 1 | | 0.966324 | | 1.016278 | | 1.023604 | |
| 26S protease regulatory subunit | | C4LY36_ENTHI | | 1 | | 0.976077 | | 1.061997 | | 0.95256 | |
| 26S protease regulatory subunit | | C4LZW8_ENTHI | | 1 | | 1.099466 | | 1.049116 | | 1.096075 | |
| 26S protease regulatory subunit | | A0A060N6S9_ENTHI | | 1 | | 0.924694 | | 0.983695 | | 0.982187 | |
| 26S protease regulatory subunit | | C4M8D7_ENTHI | | 1 | | 1.153415 | | 1.175732 | | 1.247617 | |
| 26S protease regulatory subunit | | C4M3C0_ENTHI | | 1 | | 0.976974 | | 1.040296 | | 1.050781 | |
| 26S protease regulatory subunit 7 | | B1N4Q6_ENTHI | | 1 | | 1.107603 | | 0.905793 | | 1.051844 | |
