# Supplementary material for: Single-Cell RNA Sequencing Reveals that the Switching of the Transcriptional Profiles of Cysteine-Related Genes Alters the Virulence of Entamoeba histolytica
Source: mSystems. 2020 Dec 22;5(6):e01095-20. doi: 10.1128/mSystems.01095-20 (PMC7762796; doi:10.1128/mSystems.01095-20)
Supplement: TABLE S2 [file mSystems.01095-20-st002.docx]

|  | Single cell | | | iTRAQ | | | |
| --- | --- | --- | --- | --- | --- | --- | --- |
|  | Axenic culture | CHO incubation | *In vivo* | Control | 2h with CHO | 4h with CHO | 6h with CHO |
| Hgl | 1.0000 | 0.9237 | 0.8150 | 1.0000 | 1.2845 | 1.1409 | 1.1904 |
| Igl-1 | 1.0000 | 0.8054 | 1.0384 | 1.0000 | 1.2361 | 1.0523 | 1.0442 |
| Igl-2 | 1.0000 | 0.6908 | 0.9776 | 1.0000 | 1.2617 | 1.1206 | 1.0351 |
| methionine gamma-lyase | 1.0000 | 0.6023 | 1.6642 | 1.0000 | 0.8347 | 0.7819 | 0.7411 |
| cysteine synthase | 1.0000 | 1.2056 | 0.7406 | 1.0000 | 1.0903 | 0.9481 | 1.0531 |
| Tmk65 | 1.0000 | 0.9752 | 1.1372 | 1.0000 | 1.1814 | 1.0718 | 1.0583 |
| Tmk40 | 1.0000 | 0.9430 | 0.8104 | 1.0000 | 1.0026 | 0.9512 | 0.9746 |
| Tmk03 | 1.0000 | 1.0424 | 1.2526 | 1.0000 | 1.4071 | 1.1472 | 1.4865 |
| Tmk94 | 1.0000 | 1.2225 | 0.8684 | 1.0000 | 1.1017 | 1.0104 | 1.1066 |
| Tmk37 | 1.0000 | 2.0552 | 1.0041 | 1.0000 | 1.4455 | 1.1737 | 1.5864 |
| Proteasome |  |  |  |  |  |  |  |
| EHI_005870 | 1.0000 | 1.1720 | 0.9845 | 1.0000 | 0.9792 | 0.9884 | 0.9875 |
| EHI_011870 | 1.0000 | 1.1722 | 0.9855 | 1.0000 | 1.2399 | 1.1364 | 1.2866 |
| EHI_024470 | 1.0000 | 1.1605 | 1.0601 | 1.0000 | 0.8453 | 1.0367 | 0.8583 |
| EHI_030170 | 1.0000 | 1.2930 | 0.9087 | 1.0000 | 0.9350 | 1.1387 | 1.0331 |
| EHI_053020 | 1.0000 | 1.2175 | 1.1462 | 1.0000 | 0.9770 | 1.0403 | 1.0508 |
| EHI_078710 | 1.0000 | 1.4143 | 1.1841 | 1.0000 | 1.0527 | 1.2117 | 1.0177 |
| EHI_080890 | 1.0000 | 1.1272 | 0.9342 | 1.0000 | 1.1076 | 0.9058 | 1.0518 |
| EHI_090000 | 1.0000 | 1.3608 | 1.0221 | 1.0000 | 0.9518 | 0.9870 | 1.0150 |
| EHI_103850 | 1.0000 | 1.3628 | 1.0466 | 1.0000 | 1.0213 | 1.1754 | 0.9790 |
| EHI_136180 | 1.0000 | 1.3441 | 0.9617 | 1.0000 | 1.0064 | 1.0215 | 1.0971 |
| EHI_148040 | 1.0000 | 1.3065 | 1.2629 | 1.0000 | 1.0414 | 0.9814 | 1.0935 |
| EHI_166850 | 1.0000 | 1.3513 | 1.0159 | 1.0000 | 1.1030 | 1.0919 | 1.1293 |
| EHI_174670 | 1.0000 | 1.1259 | 0.7693 | 1.0000 | 0.9668 | 1.0892 | 1.0521 |
| EHI_177320 | 1.0000 | 1.1481 | 0.8175 | 1.0000 | 0.9761 | 1.0620 | 0.9526 |
| EHI_179970 | 1.0000 | 1.4906 | 1.0391 | 1.0000 | 0.9495 | 0.9332 | 0.9773 |
| EHI_182600 | 1.0000 | 1.2987 | 1.2610 | 1.0000 | 1.1653 | 1.3110 | 1.2308 |
| EHI_185410 | 1.0000 | 1.4059 | 1.3980 | 1.0000 | 1.1534 | 1.1757 | 1.2476 |
| EHI_194570 | 1.0000 | 1.4131 | 0.6761 | 1.0000 | 1.0995 | 1.0491 | 1.0961 |
| EHI_200220 | 1.0000 | 1.0105 | 0.5907 | 1.0000 | 0.8145 | 0.9372 | 0.5839 |
