# Supplementary material for: Single-Cell RNA Sequencing Reveals that the Switching of the Transcriptional Profiles of Cysteine-Related Genes Alters the Virulence of Entamoeba histolytica
Source: mSystems. 2020 Dec 22;5(6):e01095-20. doi: 10.1128/mSystems.01095-20 (PMC7762796; doi:10.1128/mSystems.01095-20)
Supplement: TABLE S4 [file mSystems.01095-20-st004.docx]

| Gene |  | Primer sequence (5’-3’) |
| --- | --- | --- |
| Eh-Hgl | S | TGTGGTGGAGATTCTACA |
|  | AS | CATCACCAACTGCTTGAA |
| Eh-Igl-1 | S | AAAGAATGTTCATCAACTCAGCTT |
|  | AS | TTAAGCATCAGCAACCCCAACTGG |
| Eh-Igl-2 | S | TAATCTTCGCTGTTGCTTT |
|  | AS | TCAGCTCCCTTAGTGGTAA |
| Eh-actin | S | GCACTTGTTGTAGATAATGGATCAGGAATG |
|  | AS | ACCCATACCAGCCATAACTGAAACG |
| Eh-CS | S | TACATTTGGTATTGTTTATCGTGC |
|  | AS | GGTAAAAGCTCTCCAAGACCAACT |
| Eh-MGL | S | TGCTGAATTTGTGACTAGTGGA |
|  | AS | TGGATTTGGCTTCCAACACTCT |
| Eh-CP5 | S | AAAGAATGTTCATCAACTCAGCTT |
|  | AS | TTAAGCATCAGCAACCCCAACTGG |
| Eh-AP-A | S | TAATCTTCGCTGTTGCTTT |
|  | AS | TCAGCTCCCTTAGTGGTAA |
| Eh-actin | S | GCACTTGTTGTAGATAATGGATCAGGAATG |
|  | AS | ACCCATACCAGCCATAACTGAAACG |
| Eh-TMK3 | S | TACATTTGGTATTGTTTATCGTGC |
|  | AS | GGTAAAAGCTCTCCAAGACCAACT |
| Eh-TMK8 | S | TGCTGAATTTGTGACTAGTGGA |
|  | AS | TGGATTTGGCTTCCAACACTCT |
| Eh-TMK22 | S | TTTGCTTTGGAAGTGGGACATATTGTAACG |
|  | AS | TCCACGTTCCCCATTTCCATCCATTTC |
| Eh-TMK22 | S | ACACAATTGGGATATTGCCGA |
|  | AS | TGGAAAGTCCAACGAGCACT |
| Eh-TMK40 | S | TGGAGACACAGTCCATCAGA |
|  | AS | TTGTTGAAGCAGACTGATCTCT |
| Eh-TMK59 | S | AGGAAAACCAGTAAGTACAAAAGC |
|  | AS | TACTTTTTCAATTAAATGGAATCTTG |
| Eh-TMK63 | S | AAGACAACAGTAGTAGTCAGAGTT |
|  | AS | ATGTGACACACCCTGTAGGACCAG |
| Eh-TMK65 | S | TGTAGTTAATGGGTCAGGGTATAAG |
|  | AS | GCTCGTTGTTCTTCAGACATAAG |
| Eh-TMK71 | S | TGTTGAAGGAGAAAAGAAAATGAATGG |
|  | AS | CTGGTTGAGAAGGATTATGTATAGAGTC |
| Eh-TMK96 | S | AATGGGTGTGCTGTTTGTCA |
|  | AS | AAGCAACACACTTCGCGTCT |
